# Prevalence of *bla*_KPC-2_, *bla*_KPC-3_ and *bla*_KPC-30_—Carrying Plasmids in *Klebsiella pneumoniae* Isolated in a Brazilian Hospital

**DOI:** 10.3390/pathogens10030332

**Published:** 2021-03-12

**Authors:** Letícia B. Migliorini, Romário O. de Sales, Paula C. M. Koga, Andre M. Doi, Anja Poehlein, Alexandra R. Toniolo, Fernando G. Menezes, Marines D. V. Martino, Ana C. Gales, Holger Brüggemann, Patricia Severino

**Affiliations:** 1 Albert Einstein Research and Education Institute, Hospital Israelita Albert Einstein, Sao Paulo 05652-900, Brazil; lbmigliorini@gmail.com (L.B.M.); romariosallespva1@gmail.com (R.O.d.S.); 2 Laboratório Clínico, Hospital Israelita Albert Einstein, Sao Paulo 05652-900, Brazil; paula.koga@einstein.br (P.C.M.K.); andre.doi@einstein.br (A.M.D.); marines.martino@einstein.br (M.D.V.M.); 3 Department of Genomic and Applied Microbiology, Institute of Microbiology and Genetics, University of Göttingen, 37077 Göttingen, Germany; anja.poehlein@biologie.uni-goettingen.de; 4 Serviço de Controle de Infecção Hospitalar, Hospital Israelita Albert Einstein, Sao Paulo 05652-900, Brazil; alexandra.toniolo@einstein.br (A.R.T.); fernando.menezes@einstein.br (F.G.M.); 5 Department of Internal Medicine, Division of Infectious Diseases, Escola Paulista de Medicina, Universidade Federal de São Paulo, Sao Paulo 04039-032, Brazil; ana.gales@gmail.com; 6 Department of Biomedicine, Aarhus University, 8000 Aarhus, Denmark; brueggemann@biomed.au.dk

**Keywords:** antimicrobial resistance, carbapenem, healthcare-associated infection, KPC, plasmid, *Klebsiella pneumoniae*

## Abstract

*Klebsiella pneumoniae* carbapenemase (KPC) actively hydrolyzes carbapenems, antibiotics often used a last-line treatment for multidrug-resistant bacteria. KPC clinical relevance resides in its widespread dissemination. In this work, we report the genomic context of KPC coding genes *bla*_KPC-2_, *bla*_KPC-3_ and *bla*_KPC-30_ in multidrug-resistant *Klebsiella* *pneumoniae* isolates from Brazil. Plasmids harboring *bla*_KPC-3_ and *bla*_KPC-30_ were identified. Fifteen additional carbapenem-resistant *K. pneumoniae* isolates were selected from the same tertiary hospital, collected over a period of 8 years. Their genomes were sequenced in order to evaluate the prevalence and dissemination of *bla*_KPC_–harboring plasmids. We found that *bla*_KPC_ genes were mostly carried by one of two isoforms of transposon Tn*4401* (Tn*4401*a or Tn*4401*b) that were predominantly located on plasmids highly similar to the previously described plasmid pKPC_FCF3SP (IncN). The identified pKPC_FCF3SP-like plasmids carried either *bla*_KPC-2_ or *bla*_KPC-30_. Two *K. pneumoniae* isolates harbored pKpQIL-like (IncFII) plasmids, only recently identified in Brazil; one of them harbored *bla*_KPC-3_ in a Tn*4401*a transposon. Underlining the risk of horizontal spread of KPC coding genes, this study reports the prevalence of *bla*_KPC-2_ and the recent spread of *bla*_KPC-3,_ and *bla*_KPC-30_, in association with different isoforms of Tn*4401*, together with high synteny of plasmid backbones among isolates studied here and in comparison with previous reports.

## 1. Introduction

Infections caused by carbapenem-resistant *Enterobacteriaceae* represent a significant global health threat [1]. *Enterobacteriaceae* become resistant to carbapenems by three major mechanisms: porin alteration, hyper-expression of efflux pumps, and β-lactamase production [2]. There are three main groups of β-lactamases associated with resistance to carbapenems (carbapenemases): *Klebsiella pneumoniae* carbapenemase (KPC), metallo-β-lactamases, and OXA-48-like β-lactamase. Mobile genetic elements, such as plasmids and transposons, are the main vectors enabling and facilitating the spread of these resistance determinants [3,4].

KPC is a plasmid-encoded enzyme which occurs in several different variants, and its clinical relevance is associated with its worldwide spread [5]. The occurrence of carbapanemases varies according to the geographic region; the KPC-2 variant has been reported as the most frequent carbapenemase in Latin America [6,7]. In Brazil, the first report of KPC-2 occurred in 2006, and soon, numerous reports attested to its widespread dissemination [8,9,10,11]. KPC-2 and KPC-3 differ by only one amino acid (H272Y) and are the most prevalent carbapenemases worldwide [12,13,14,15]. Only KPC-2 is currently considered endemic in Brazil [10,16]. The treatment of infections by carbapenem-resistant *Enterobacteriaceae* is complicated, with severe clinical and socioeconomic consequences [4,17,18].

Despite the worldwide spread of KPC, genomic studies including KPC-harboring isolates from Latin America, and in particular from Brazil, are underrepresented in the literature. For instance, a recent study evaluated all KPC-harboring plasmids described in the NCBI database, and only 12 out of 257 representative KPC-plasmids evaluated were from Brazil, all harboring *bla*_KPC-2_ [19].

Our hospital is one of the Brazilian medical centers enrolled in a longitudinal surveillance study (Study for Monitoring Antimicrobial Resistance Trends, SMART) [20]. Due to our participation, two KPC variants not previously reported in *Enterobacteriaceae* in Brazil, *bla*_KPC-3_, and *bla*_KPC-30_, were identified in *K. pneumoniae* isolated in Brazil [21]. Both isolates showed resistance to colistin, a last-resort therapeutic drug against multidrug-resistant *Enterobacteriaceae*.

In this work, we report the genomic characterization of these two isolates by whole genome sequencing. In addition, 15 *K. pneumoniae* isolates, collected in the same tertiary hospital over a period of 8 years, were genome-sequenced. We found *bla*_KPC_-harboring plasmids in most isolates; these originated from two different plasmids and contained *bla*_KPC-2_, *bla*_KPC-3_, or *bla*_KPC-30_, mostly on one of two isoforms of transposon Tn*4401* (Tn*4401*a or Tn*4401*b).

## 2. Results

Genome characteristics and MLST types for the *K. pneumoniae* isolates harboring *bla*_KPC-2_, *bla*_KPC-30_, and *bla*_KPC-3_ are summarized in Table 1. All isolates belonged to the Clonal Complex 258 (CC258), except for Kp326 (ST16). Capsule polysaccharide synthesis (cps) locus analysis demonstrated that isolates belonged to five distinct K-loci groups, the most recurrent being KL36, as previously reported [22].

**Figure 1:**
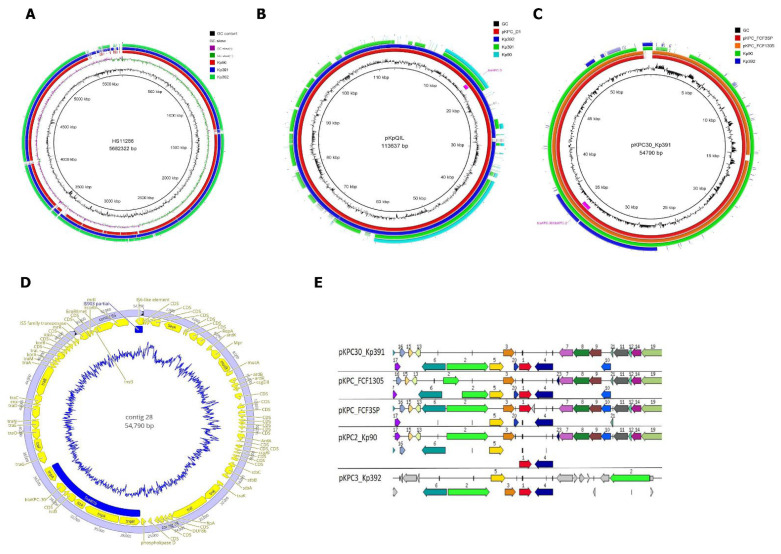
Genomic characteristics of *Klebsiella pneumoniae* isolates. (**A**) Genome comparison of three *Klebsiella pneumoniae* isolates using strain HS11286 as the reference genome. Genome sizes were similar to the reference genome *K. pneumoniae* strain HS11286 (5682 kb) and differences are mostly associated with acquired genomic regions. (**B**) The plasmid in strain Kp392 is highly similar to plasmids pKpQIL and pKPC_D1. The analysis compared the plasmid pKpQIL with the plasmid pKPC_D1 and the genomes of the three strains sequenced here. Only the genome of strain Kp392 contains contigs (including the 21 kb contig that carries *bla*_KPC-3_) that match almost entirely the plasmid pKpQIL. The strain Kp391 carries a different plasmid that shares some homology with pKpQIL. The *bla*_KPC-3_ gene is highlighted in pink. (**C**) The *bla*_KPC-30_ carrying plasmid pKPC30_Kp391 in strain Kp391 is highly similar to *bla*_KPC-2_ carrying plasmids pKPC_FCF3SP and pKPC_FCF1305. The analysis compared the assembled plasmid sequence in strain Kp391 with the plasmids pKPC_FCF3SP and pKPC_FCF1305 and the genomes of the other strains sequenced here (Kp90 and Kp392). pKPC30_Kp391 almost entirely matches the plasmids pKPC_FCF3SP and pKPC_FCF1305. The strain Kp90 also carries a *bla*_KPC-2_ encoding plasmid that shares high homology with pKPC30_Kp391, pKPC_FCF3SP and pKPC_FCF1305. The location of *bla*_KPC-2_ or *bla*_KPC-30_ is highlighted in pink. (**D**) General structure of pKPC30_Kp391 based on the IncN conserved backbone and the two acquired regions Tn*4401b* and IS903B. The conserved lncN region carries genes involved in replication and replication regulation (*repA*, *ardB*, *ardR*, *ardK*, *ccgEIII*, *ccgD*, *ccgC*, *ccgAI*), genes responsible for plasmid stability (*stbA*, *stbB*, *stbC*, *fipA*, *eex*, *korA*, *korB*, *kikA*), in DNA repair (*mpr*, *mucA*, *mucB*), inhibition of type I restriction enzymes (ardA), and conjugative transfer (*tra* gene region). Transposon Tn*4401b* comprises *tnpR*, *tnpA*, *istA*, *istB*, *bla*_KPC_, and *tnpA*. (**E**) Plasmid gene organization in the vicinity of *bla*_KPC_ gene in pKPC_Kp90, pKPC_Kp391, pKPC_Kp392, and closely related plasmids. The colors indicate high homology of the genes. 1, *bla*_KPC_ gene; 2 (*tnpA*) and 4 (*tnpA*) (transposase); 3 (*istB*) and 5 (*istA*) (mobile element); 6 (*tnpR*) site-specific recombinase; 7–14 (*tra* genes) components of a type IV conjugative transfer system; 15–17, hypothetical proteins.

All isolates harbored the *bla*_KPC-2_ gene, except for Kp391, harboring *bla*_KPC-30_, and Kp392, harboring *bla*_KPC-3_. We selected three isolates harboring distinct *bla*_KPC_ variants for a complete genomic characterization: Kp90 (*bla*_KPC-2_), Kp391 (*bla*_KPC-30_), and Kp392 (*bla*_KPC-3_). Major differences between the three genomes are depicted in Figure 1A. We then looked for the genomic loci of the KPC genes; in all strains, the genes were located on contigs that were parts of plasmids (Figure 1B). This is in agreement with previous observations, that is, the *bla*_KPC_ gene is mostly carried by a multireplicon IncFIIk-IncFI plasmid [23,24]. In fact, in Kp392, the *bla*_KPC-3_ gene was located on a region that is 100% identical to a region of the plasmid pKpQIL (113,637 bp) found in *K. pneumoniae* ST258 strains [25] and to a region of plasmid pKPC (113,639 bp) found in a ST512 strain [26] (Figure 1B). Kp392 from this work is also a ST512 strain, and the plasmid in strain Kp392 is a multireplicon IncFIIk-IncFI plasmid (Figure 1B). Next, we looked into the *bla*_KPC-30_-harboring strain Kp391 that belongs to the well-known ST11 [27,28,29,30]. The *bla*_KPC-30_ gene was located on a 49 kb contig that showed high similarity with the 54 kb plasmid pKPC_FCF3SP that carries *bla*_KPC-2_ in strain FCF3SP (ST442) isolated from a blood culture of a patient in Brazil [31]. The entire plasmid in strain Kp391 could be reconstructed using pKPC_FCF3SP as template. The assembled plasmid is designated pKPC30_Kp391 (Figure 1C). All genes present in the lncN plasmid backbone were found in pKPC30_Kp391 (Figure 1D). The plasmids from strains FCF3SP and Kp391 share 99% nucleotide identity, with a total of 76 SNPs. The strain Kp90 carries a *bla*_KPC-2_-encoding plasmid that shares high similarity with pKPC30_Kp391, pKPC_FCF3SP, as well as pKPC_FCF1305. Figure 1E describes the genomic region of the three plasmids.

**Figure 2:**
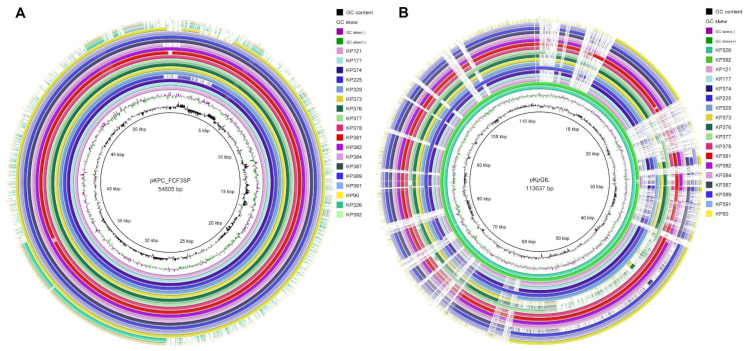
Presence of plasmids in 17 sequenced *Klebsiella pneumoniae* isolates. (**A**) Comparison using pKPC_FCF3SP as the reference plasmid identified 15 strains that contained highly identical plasmids. (**B**) A comparison using pKpQIL as the reference plasmid identified two strains (Kp326 and Kp392) with highly similar plasmids.

Fourteen additional *K. pneumoniae* isolates that were collected in the same tertiary hospital over a period of 8 years were sequenced and analyzed. We searched for the genomic region where the *bla*_KPC_ genes were located in these 14 strains; in all strains, the genes were located on contigs that were parts of plasmids belonging to two distinct incompatibility groups: lncN or lncFII. Among the 17 sequenced isolates, 15 harbored the lncN backbone, showing high similarity with pKPC_FCF3SP (Figure 2A). On this plasmid, except for Kp391, harboring *bla*_KPC-30_, all isolates harbored *bla*_KPC-2_. Isolates Kp326 (*bla*_KPC-2_) and Kp392 (*bla*_KPC-3_) contained plasmids highly similar to the plasmid pKpQIL (lncFII) (Figure 2B). pQIL-like (IncFIB) plasmids have just recently been reported in Brazil, in clones belonging to ST16, such as Kp326, and associated with high mortality rates [32]. However, the sequence of the pQIL-like plasmid identified in Brazil is not publicly available, so we were not able to compare ours to it and, to our knowledge, the first sequence made publicly available.

**Figure 3:**
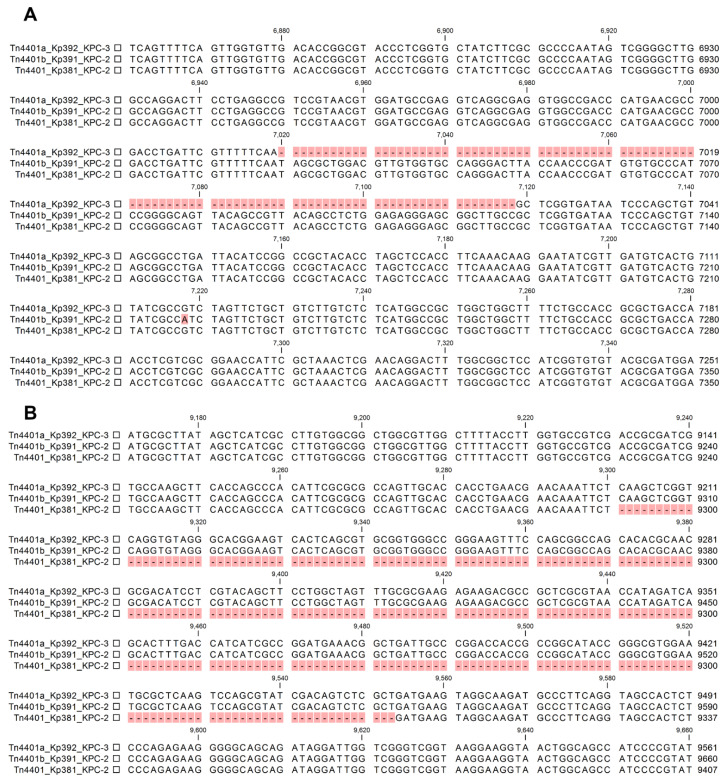
Comparison of the three Tn*4401* isoforms found in *K. pneumoniae* isolates in this work. (**A**) Tn*4401*a_Kp392 shows a deletion of 100 bp in the region upstream of the *bla*_KPC_ gene when compared with Tn*4401*b_Kp391 and Tn*4401*_Kp381. (**B**) Downstream from the *bla*_KPC_, gene Tn*4401*_Kp381 shows a deletion of 253 bp when compared with Tn*4401*a_Kp392 and Tn*4401*b_Kp391.

Three isoforms of the Tn*4401* transposon were found in the isolates described in this study. Two isoforms are well-known, Tn*4401*a and Tn*4401*b. These isoforms differ by a 100 bp deletion in the region upstream of *bla*_KPC_ in Tn*4401*a (Figure 3A). The isoform Tn*4401*a was carried by pKpQIL-like plasmids (strains Kp326 and Kp392), while the isoform Tn*4401*b was carried by pKPC_FCF3SP-like plasmids, as previously reported [31]. It has been shown that isolates harboring Tn*4401*a present higher resistance to carbapenems due to alterations in the promoter region of *bla*_KPC_ [33,34]. A third isoform of Tn*4401* was detected in isolate Kp381. This variant lacks 253 bp downstream from the *bla*_KPC_ gene, leading to a shorter *tnpA/ISKpn6* region (1320 bp/439 aa to 963 bp/320 aa) (Figure 3B).

As a means to understand the impact of the different *bla*_KPC_ genes in the susceptibility profile of *K. pneumoniae*, the susceptibility profiles of Kp391 (*bla*_KPC-30_), Kp392 (*bla*_KPC-3_), and Kp90 (*bla*_KPC-2_) are reported in Table 2. All three isolates showed resistance to cephalosporins, quinolones, colistin, and carbapenems, and were susceptible to the combination ceftazidime/avibactam. Kp392, harboring the pKpQIL-like plasmid with Tn*4401*a and *bla*_KPC-3_, displayed a MIC of 6 µg/mL for the combination ceftazidime/avibactam (breakpoint for resistance: ≥8 µg/mL); this is higher than for the other two isolates which were clearly susceptible (2 µg/mL) [35].

## 3. Discussion

KPC-producing *Enterobacteriaceae* have been considered a pandemic in the history of Gram-negative bacteria [37]. According to gene sequences deposited in Genbank (https://www.ncbi.nlm.nih.gov/pathogens/refgene/#kpc, accessed on 4 February 2021) at the time of this submission (date: 4 February 2021), a total of 66 *bla*_KPC_ gene variants had been reported.

As a result of the antibiotic resistance surveillance study, SMART, two new KPC variants were detected in *K. pneumoniae* in Brazil: *bla*_KPC-3_ and *bla*_KPC-30_ [21]. This work was conducted to gain an understanding of the genetic context surrounding *bla_KPC_* genes in the tertiary hospital where the new variants were detected, using this information to discuss the scenario in Brazil.

KPC-3, still not endemic in Brazil, differs by one amino acid substitution (H272Y) from KPC-2 [38]. This single amino acid change has been reported to increase the catalytic efficiency of the enzyme by up to nine times when degrading ceftazidime [14], and, most recently, it has been described to increase the MIC for ceftazidime/avibactam [14,15,39]. This is particularly worrisome since, in Brazil, this combination has only recently been approved as a treatment option for multidrug-resistant bacteria (June 2018). Strains Kp90 (harboring *bla*_KPC-2_), Kp391 (harboring *bla*_KPC-30_), and Kp392 (harboring *bla*_KPC-3_) showed very similar susceptibility profiles for carbapenems and ceftazidime. However, Kp392 displayed a MIC of 6 µg/mL for ceftazidime/avibactam (breakpoint for resistance: ≥8 µg/mL) [35], compared to Kp90 and Kp391, which were clearly susceptible (2 µg/mL). We suggest that the increased resistance to ceftazidime/avibactam found for Kp392 (MIC of 6 mg/mL) is mostly associated with the presence of KPC-3, in agreement with previous studies [14,40]. Additionally, in Kp392, the pKpQIL-like plasmid harbored the *bla*_KPC-3_ gene in a Tn*4401*a transposon; this transposon has been associated with increased resistance to carbapenems due to modifications in the promoter region that increase the expression of the *bla*_KPC-3_ gene (a 100 bp deletion upstream of this gene) [33,34].

Plasmid pKpQIL belongs to the lncFII incompatibility group and has often been reported in association with antibiotic resistance and epidemic isolates. It was first reported in Israel in 2006, but a retrospective study revealed that it had been carried by a *K. pneumoniae* isolate from a patient in New York in 2004 [41,42,43,44]. This plasmid was the first *bla*_KPC_-bearing plasmid identified in epidemic ST258 strains, becoming well-known for the early dissemination of KPC-encoding genes [25,45,46]. Despite its worldwide spread, pKpQIL-like plasmids were only recently reported in Brazil in *K. pneumoniae* ST16 [25,32,41,43,44,47,48]. In this work, isolates harboring this plasmid were collected in 2016 and 2017 in strains belonging to ST16 and ST512, suggesting a recent, and possibly local, spread.

Plasmids belonging to IncN, such as pKPC_FCF3SP, and highly similar plasmids identified in this study (in strains isolated since 2011), have been reported in Brazil since 2005; the earliest report of an IncN plasmid carrying *bla*_KPC-**2**_ was in 1997 [19,31,49]. Even though pKPC_FCF3SP is associated with the *bla*_KPC-2_ gene, here we report an isolate carrying a pKPC_FCF3SP-like plasmid with *bla*_KPC-30_. The *bla*_KPC-30_ gene sequence has been previously reported in one strain from Brazil, but without any associated publication (strain 1472816, GenBank accession number KY646302.1). The *bla*_KPC-30_ variant shows a single-point mutation compared to *bla*_KPC-2_, leading to one amino acid change (R6H) in the region coding for the signal peptide. In this regard, a high degree of synteny between *bla*_KPC-2_ and *bla*_KPC-30_-carrying plasmids was noted and, as matter of fact, highly similar plasmids have often been identified in epidemic strains, such as those belonging to CC258 [45,50,51].

In conclusion, in this work we reported the genetic background for *bla*_KPC_ found in carbapenem-resistant *K. pneumoniae* isolates from Brazil. Plasmids highly similar to pKpQIL and pKPC_FCF3SP harbored *bla*_KPC-2,_ *bla*_KPC-3_, *bla*_KPC-30_, in association with different isoforms of Tn*4401*, an active transposon encoding insertion sequences (ISKpn7 and ISKpn6) capable of efficiently mobilizing the *bla*_KPC_ gene to random targets. The diversity and structural complexity of genetic elements carrying *bla*_KPC_ genes suggest that they play major roles in actively promoting transposition of these genes to various genetic locations in the bacterial genome, but also suggest that they may increase the genetic plasticity of plasmids, leading to improved ability to coexist with bacterial hosts [45]. However, we also observed high synteny of plasmid backbones among isolates studied here and in comparison with previous reports from Brazil and the rest of the world. This highlights the importance of surveillance for early detection and implementation of control measures to prevent the rapid dissemination of *bla*_KPC_ in the clinical environment.

## 4. Materials and Methods

### 4.1. Clinical Isolates

*K. pneumoniae* isolates harboring *bla*_KPC_ genes were identified as *K. pneumoniae* by MALDI-TOF MS (Bruker Daltonics, Billerica, MA, USA) between 2011 and 2017. All patients had hospital-acquired infections, with a history of previous hospitalization and antimicrobial use (carbapenem, quinolone, cephalosporin, or piperacillin-tazobactam) during the 30 days prior to *K. pneumoniae* isolation.

### 4.2. Antibiotic Susceptibility Testing

Antibiotic susceptibility was determined using the Vitek 2 XL System (bioMérieux, Craponne, France). Additionally, susceptibility to ceftazidime/avibactam, imipenem, and meropenem was also carried out by the epsilometric (Etest^®^) method. Broth microdilution was performed for MIC testing of polymyxin B with *Pseudomonas aeruginosa* ATCC 27853 used as a reference for susceptibility. Antimicrobial susceptibility results were interpreted according to BrCAST/EUCAST guidelines [52].

### 4.3. Genome Sequencing

Genomic DNA isolation of *K. pneumoniae* isolates was performed as previously described [53]. Concentration and purity of the isolated DNA was first checked with a NanoDrop ND-1000 (Peqlab, Erlangen, Germany), and the exact concentration was determined using the Qubit^®^ dsDNA HS Assay Kit, as recommended by the manufacturer (Life Technologies GmbH, Darmstadt, Germany). Illumina shotgun libraries were prepared using the Nextera XT DNA Sample Preparation Kit and subsequently sequenced on a MiSeq system using the reagent kit v3 with 600 cycles (Illumina, San Diego, CA, USA), as recommended by the manufacturer. Quality filtering was done with version 0.36 of Trimmomatic [54]. Assembly was performed using the SPAdes genome assembler software version 3.13.0 [55], using an average of 2,009,581 paired-end reads (range: 1,716,540–2,544,588). Qualimap version 2.2.1 [56] was used to validate the genome assembly and determine the sequence coverage. The average coverage was 81-fold (range: 69–103). Comparative genome and plasmid analyses were done using RAST and visualized with BRIG [57]. Insertion sequences were identified by IS finder [58].

### 4.4. MLST and Capsule Synthesis Loci (K-loci) Analysis

WGS data were also used to determine the sequence types (STs) using the multi-locus sequencing typing (MLST) scheme available at (https://bigsdb.pasteur.fr/cgi-bin/bigsdb/bigsdb.pl?db=pubmlst_klebsiella_seqdef&page=sequenceQuery/; accessed on 2 April 2020) sited at the Institut Pasteur MLST. Capsule synthesis loci (K-loci or KL) analysis was carried out using the software Kaptive available at http://kaptive.holtlab.net/, accessed on 2 April 2020 [59].

### 4.5. NCBI Database Data Extraction for K. pneumoniae KPC-Harboring Plasmids

The KPC-2, -3, and -30 sequences (Accession Numbers NC_019161.1, NG_049257.1 and NG_054685.1) were used for a nucleotide-nucleotide BLAST search in the NCBI database (expected threshold 10 × 10^−70^), following the protocol described by Brandt et al. [19]. The resulting sequence hits (accessed on February 2021) were then filtered for sequences reported only in *K. pneumoniae* and for plasmid structures (with at least 1000 bp) carrying KPC-2, -3, and -30 nucleotide sequences.

## Figures and Tables

**Table 1:** Features of sequenced genomes of *K. pneumoniae*.

Isolate	Year of Isolation	Origin of Specimen	*bla-*_KPC_ Variant	Mlst Type (ST)	Clonal Group (CG)	CPS *Locus* (KL)	Genome Size (kb)	G+C (%)	Coverage (Fold)	Contigs (n ^1^)	CDS (n)	Genbank Accession
Kp90	2015	*Blood*	*bla-*_KPC-2_	ST437	258	36	5429	57.4	77	76	5496	JACBOR000000000
Kp391	2016	*Abdominal abscess*	*bla-*_KPC-30_	ST11	258	64	5871	56.9	77	67	5956	JACBOQ000000000
Kp392	2017	*Urine*	*bla-*_KPC-3_	ST512	258	107	5681	57.1	103	81	5787	JACBOP000000000
Kp121	2014	*Blood*	*bla-*_KPC-2_	ST437	258	36	5554	57.3	84	55	5601	JAEVGO000000000
Kp177	2013	*Blood*	*bla-*_KPC-2_	ST437	258	36	5588	57.3	83	79	5679	JAEVGN000000000
Kp225	2011	*Blood*	*bla-*_KPC-2_	ST437	258	36	5777	57.1	64	81	5847	JAEVGM000000000
Kp326	2016	*Blood*	*bla-*_KPC-2_	ST16	-	51	5568	57.2	91	73	5648	JAEVGL000000000
Kp329	2015	*Bronchoalveolar lavage*	*bla-*_KPC-2_	ST11	258	64	5887	57.1	66	127	6049	JAEVGK000000000
Kp384	2014	*Blood*	*bla-*_KPC-2_	ST437	258	36	5569	57.3	66	79	5672	JAEVGJ000000000
Kp387	2013	*A*scitic fluid	*bla-*_KPC-2_	ST11	258	27	5852	57.0	51	105	5967	JAEVGI000000000
Kp389	2013	*Bronchoalveolar lavage*	*bla-*_KPC-2_	ST437	258	36	5473	57.3	88	75	5524	JAEVGH000000000
Kp373	2013	Rectal *surveillance* swabs	*bla-*_KPC-2_	ST11	258	64	5900	57.0	81	68	6021	JAEVGG000000000
Kp374	2013	Rectal *surveillance* swabs	*bla-*_KPC-2_	ST437	258	36	5604	57.3	84	78	5702	JAEVGF000000000
Kp376	2015	Rectal *surveillance* swabs	*bla-*_KPC-2_	ST11	258	27	6162	56.6	82	135	6385	JAEVGE000000000
Kp377	2015	Rectal *surveillance* swabs	*bla-*_KPC-2_	ST11	258	64	5808	56.8	91	115	5953	JAEVGD000000000
Kp378	2016	Rectal *surveillance* swabs	*bla-*_KPC-2_	ST11	258	64	5872	56.9	87	62	5965	JAEVGC000000000
Kp381	2017	Rectal *surveillance* swabs	*bla-*_KPC-2_	ST437	258	36	6095	56.5	78	128	6344	JAEVGB000000000
Kp382	2017	Rectal *surveillance* swabs	*bla-*_KPC-2_	ST11	258	64	5877	56.9	94	97	6023	JAEVGA000000000

^1^ N: number.

**Table 2:** Antibiotic susceptibility of three *K. pneumoniae* isolates with different *bla*_KPC_ genes.

	Kp90		Kp391		Kp392	
Antimicrobial	MIC µg/mL	Profile	MIC µg/mL	Profile	MIC µg/mL	Profile
Ampicillin/Sulbactam	≥32	R	≥32	R	≥32	R
Piperacilin/Tazobactam	≥128	R	≥128	R	≥128	R
Cefuroxime	≥64	R	≥64	R	≥64	R
Cefoxitin	≥64	R	≥64	R	≥64	R
Ceftazidime	16	R	≥64	R	≥64	R
Ceftriaxone	≥64	R	≥64	R	≥64	R
Cefepime	≥64	R	≥64	R	≥64	R
Ertapenem	≥8	R	≥8	R	≥8	R
Imipenem	≥16	R	≥16	R	≥16	R
Meropenem	≥16	R	≥16	R	≥16	R
Amikacin	≤2	S	≤2	S	≥64	R
Gentamicin	≤1	S	≤1	S	8	R
Ciprofloxacin	≥4	R	≥4	R	≥4	R
Tigecycline	≥8	-	≤0.5	-	2	-
Imipenem	>32	R	>32	R	32	R
Meropenem	>32	R	>32	R	>32	R
Ceftazidime/avibactam	2	S	2	S	6	S
Colistin	≥16	R	≥16	R	≥16	R

**MIC:** minimum inhibitory concentration. Breakpoints for Tigecycline are not available for *K. pneumoniae* [36].

## Data Availability

All genome sequences were deposited in the GenBank with the following accession numbers: JACBOR000000000, JACBOQ000000000, JACBOP000000000, JAEVGO000000000, JAEVGN000000000, JAEVGM000000000, JAEVGL000000000, JAEVGK000000000, JAEVGJ000000000, JAEVGI000000000, JAEVGH000000000, JAEVGG000000000, JAEVGF000000000, JAEVGE000000000, JAEVGD000000000, JAEVGC000000000, JAEVGB000000000, JAEVGA000000000.
